# Delineating the Significance of Several Inflammatory Markers in a Lung Tuberculosis Cohort During the Active and Post-Tuberculosis Stages of the Disease: An Observational Study in Cape Town, South Africa (2019 to 2024)

**DOI:** 10.3390/idr17030052

**Published:** 2025-05-09

**Authors:** Chrisstoffel Jumaar, Lindiwe Malefane, Steve Jacobs, Olakunle Sanni, Elize Louw, Nicola Baines, Carmen Payne, Sigrid Schulz, Carl Lombard, Merga Feyasa, David Maree, Shantal Windvogel, Hans Strijdom, Benjamin Botha, Brian Allwood, Gerald J. Maarman

**Affiliations:** 1 Centre for Cardio-Metabolic Research in Africa, Division of Medical Physiology, Department of Biomedical Sciences, Faculty of Medicine & Health Sciences, Stellenbosch University, Cape Town 8000, South Africa; 27421309@sun.ac.za (C.J.); 27583708@sun.ac.za (L.M.); stevejacobs@sun.ac.za (S.J.); kunlesola2810@gmail.com (O.S.); shantalw@sun.ac.za (S.W.); jgstr@sun.ac.za (H.S.); 2 Division of Pulmonology, Department of Medicine, Faculty of Medicine & Health Sciences, Stellenbosch University & Tygerberg Hospital, Cape Town 8000, South Africa; ehlouw@sun.ac.za (E.L.); nicolabaines@sun.ac.za (N.B.); sigrid.schulz@westerncape.gov.za (S.S.); david.maree@westerncape.gov.za (D.M.); brianallwood@sun.ac.za (B.A.); 3 Biostatistics Research Unit, South African Medical Research Council, Cape Town 8000, South Africa; cjlombard@sun.ac.za; 4 CDT—Africa Center of Excellence, College of Health Sciences, Addis Ababa University, Addis Ababa 10001, Ethiopia; merga.belina@aau.edu.et; 5 Division of Epidemiology and Biostatistics, Department of Global Health, Stellenbosch University, Cape Town 8000, South Africa; 6 Cape Winelands TB Centre, Brewelskloof Hospital, Worcester 6850, South Africa; benjamin.botha@westerncape.gov.za

**Keywords:** tuberculosis, post-TB lung disease, inflammatory pathways, inflammation

## Abstract

Background: Pulmonary tuberculosis (TB) frequently leads to long-term lung complications that contribute to increased mortality. Understanding the pathogenesis of post-TB lung impairments is crucial for improving long-term outcomes in TB patients; yet this area remains poorly researched. Methods: Our study assessed circulatory inflammatory markers in patients who completed TB treatment more than one year before enrolment (population 1) and patients receiving in-hospital treatment for active drug-sensitive TB (population 2). Results: IL-6 was seven times higher in both populations compared to the normal range. IL-8 was below the limit of detection (LOD) in population 1, while it was approximately 2.5 times higher in population 2 compared to the normal range. TNF-α was 21 times higher in population 1 and 19 times higher in population 2 compared to the normal range. CRP was almost 49 times higher in both populations, and IL-1Ra was below the LOD in population 1, while it was ~1.5 times higher in population 2 compared to the normal range. Conclusions: These inflammatory biomarkers correlated well with lung function in the post-TB state, and their high levels suggest a persistent pro-inflammatory state post-TB, which may contribute to post-TB lung disease. More research is warranted to better understand this phenomenon, but these findings may highlight a need to consider anti-inflammatory therapy for patients with post-TB lung disease, especially since these high levels of cytokines can directly contribute to lung damage.

## 1. Introduction

Pulmonary tuberculosis (TB) is a serious airborne lung infectious disease. In 2022, the highest burden of the disease occurred in adult males, accounting for 55% of all cases, while women and children accounted for the remaining 45% [1]. Although *Mycobacterium tuberculosis* (MTB) was first identified as the cause of TB centuries ago, it remains a major contributor to global mortality rates [2]. According to the 2023 Global Tuberculosis Report, TB is the second leading cause of death worldwide by an infectious agent [1]. TB transmission is influenced by various factors, including the concentration of bacilli inhaled, ventilation, proximity, and duration of exposure [3]. Thus, the TB pandemic is complex and is further influenced by factors such as poverty and HIV/AIDS [4]. In 2022, TB claimed 1.3 million lives globally, almost double the deaths caused by HIV/AIDS [1]. This highlights the need for improved prevention, diagnosis, and treatment strategies to combat the spread of TB and reduce its impact on global health [4].

Notably, the estimated global prevalence of TB reached 10.6 million cases in 2022, with an alarming 7.5 million newly diagnosed cases, marking the highest number since 1995. Despite successful TB treatment, some patients develop significant post-TB lung impairments [5]. Changes in parenchymal and bronchial structures during TB infection may be irreversible and result in permanent restriction or obstruction of airflow [6]. Studies in Denmark and the United Kingdom have reported higher mortality rates in patients who are grouped as post-TB compared to the general population [7,8].

Consequently, the period between active TB and clinical presentation of post-TB lung impairments is crucial in the TB continuum. This highlights the importance of researching therapeutic strategies to prevent lasting lung impairment. However, the pathophysiological events during this period are not well understood. Some studies have hypothesized that a pro-inflammatory state might be the key driver in the pathogenesis of post-TB lung impairments [5,9,10]. Despite this, a notable gap exists, and we believe that a study aiming to map the various inflammatory pathways in active TB compared to post-TB could make timely contributions to the field. Therefore, the present study aims to delineate the significance of several inflammatory markers in this clinical context, hoping to fill in the gaps in the literature.

## 2. Materials and Methods

### 2.1. Study Design

This study was nested within a larger clinical study, the PuPPeT II trial [11] in Cape Town, South Africa, a cross-sectional study (from 2019 to 2024) that evaluates the prevalence of pulmonary hypertension across different TB patient populations. For this sub-study, two patient populations were considered: population 1 (comprising patients who have completed TB treatment more than one year before enrolment) and population 2 (comprising patients receiving in-hospital TB treatment at the time of data collection). The rationale here was to investigate if time (measured in years) after the completion of TB treatment is a period where physiological changes may occur that could predispose to TB sequelae or whether current drug-sensitive TB that is clinically managed would contribute to an existing pro-inflammatory state. Ethical approval was obtained from Stellenbosch University’s Human Research Ethics Committee (N18/08/091, approved on 1 November 2018).

### 2.2. Phlebotomy

Each patient’s blood was collected in 5 mL plasma and serum tubes (PLAS-GD100EK2 and PLAS-VAC-Y5, Gongdong Medical Technology Co., Ltd, Riverside, CA 92507, USA). Plasma collection tubes were mixed thoroughly and contained the anticoagulant ethylenediaminetetraacetic acid that blocks the coagulation cascade by binding to calcium ions. Transportation of blood to the laboratory took approximately 90 min. All tubes were centrifuged at 3000 rpm for 10 min in a Biosafety Cabinet (BSC) Class II (Centrifugal fan H14 HEPA filter) (BIOBASE, Shandong, China) using a centrifuge (5810, Merck Eppendorf^®^, Cape Town, South Africa). Serum collection tubes (VacuLab^®^ SSGT tubes, Jiangsu, China) were coated with a clot activator and gel, allowing for serum separation, and were left to clot before being placed on ice. Samples were stored as 50 μL aliquots at −80 °C, and all blood was handled according to the Centers for Disease Control and Prevention guidelines [12].

### 2.3. Measuring Serum Inflammatory Markers with ELISA

Serum levels of IL-6, IL-8, TNF-α, and high-sensitivity CRP were quantified using ELISA kits from Elabscience, with the respective catalog numbers: E-EL-H6156, CSB-E04641h, E-EL-H0109, and RK00078. Serum IL-1Ra was quantified using the Simple Step ELISA kit (Catalog No: ab211650). Serum markers were chosen based on various factors, including those previously identified in a TB context, feasibility, and financial restraints. All samples were adequately stored at −80 °C and were used for assays less than 3 years after collection. All inflammatory markers were measured according to the manufacturer’s instructions. Serum samples and standards were added to 96-well ELISA microplates pre-coated with antibodies specific to the respective human inflammatory marker. After incubation at 37 °C, biotin antibody was added and incubated. The plates were then washed to remove unbound antigens. Subsequently, horseradish peroxidase-avidin or conjugate solution was added and incubated, followed by another wash step. Then, 3,3′,5,5′-tetramethylbenzidine substrate was added and incubated at 37 °C. The absorbance was measured at 450 nm using the microplate reader (FDA 21 CFR Part 11, BMG Labtech, Ortenberg, Germany). Inflammatory marker concentrations in the samples were extrapolated from the standard curve. Samples were measured in duplicate, and the mean value was used for analysis.

### 2.4. Statistics

Statistical analyses were performed using Stata 16 (StataCorp (2019) Stata Statistical Software: Release 16. StataCorp LLC, College Station, TX, USA) and GraphPad Prism 10.2 (GraphPad Software. Inc, Boston MA, USA) by statisticians at the Faculty of Medicine and Health Sciences of Stellenbosch University. A subset of patients from two study populations was randomly selected from the main study. A power analysis was performed to determine the number of patients required from each population to maintain statistical power. The analysis utilized the parameters: alpha = 0.0500, power = 0.8000, delta = 0.4000, N_g = 4, Var_m = 0.1600, Var_e = 1.0000. The analysis confirmed that a sample size of n = 21–22 in each population would hold statistical power. Statistical significance was considered at *p* < 0.05. The data underwent normality testing using the Shapiro–Wilk test. If the data were normal, a parametric *t*-test was performed, and if the data were not normally distributed, a non-parametric *t*-test was used. Descriptive statistics included the mean, standard deviation, and standard error of the mean. Pearson’s correlation coefficient was used to correlate the inflammatory biomarkers with clinical endpoints like lung function parameters.

## 3. Results

### 3.1. Patient Demographic Data

In terms of age, there was a relatively equal distribution of patients across the different age brackets (18–60 and above), while there were slightly more males than females. In population 1, 68% were current smokers, and in population 2, 43% were smokers. The majority of both populations were HIV-negative, and those who were positive were on treatment. Whereas most patients did not have heart disease (Table 1).

### 3.2. Clinical Data

Regarding the clinical data, in population 1, 50% of the patients had only one previous TB episode, and 50% had two. In population 2, 38% of the patients had no previous TB episodes, whereas most had at least one previous TB episode. In population 1, the mean lung function was better than that of patients from population 2 when comparing the spirometry parameters FVC % predicted and FEV 1% predicted (Table 2).

### 3.3. Inflammatory Markers

In terms of the inflammatory markers, there were no differences in these markers between the two populations. However, compared to the normal range, IL-6 was higher in both populations, IL-8 was below the LOD in population 1, while it was higher in population 2 compared to the normal range (Table 3). TNF-α and CRP were higher in both populations, while IL-1Ra was below the LOD in population 1 and higher in population 2 compared to the normal range (Table 3).

### 3.4. Correlation of Cytokines with Clinical Endpoints

**Figure 1:**
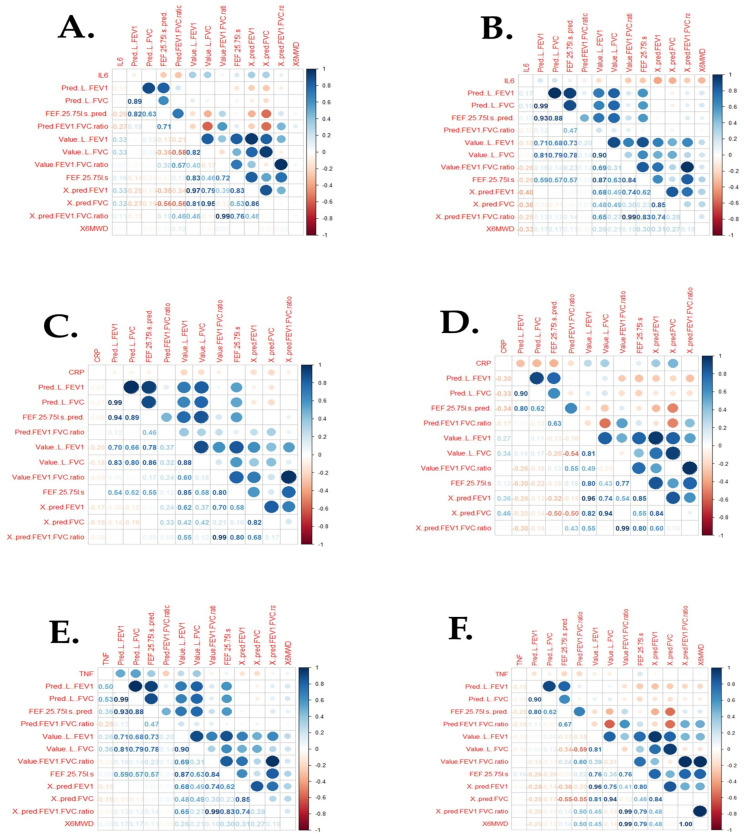
Pearson correlations between inflammatory markers and clinical lung function parameters. (**A**) correlations between IL-6 and clinical endpoints of population 1; (**B**) correlations between IL-6 and clinical endpoints of population 2; (**C**) correlations between CRP and clinical endpoints of population 1; (**D**) correlations between CRP and clinical endpoints of population 2; (**E**) correlations between TNF-alpha and clinical endpoints of population 1; (**F**) correlations between TNF-alpha and clinical endpoints of population 2.

In population 1, CRP seems to have a weak linear negative relationship with all the lung function parameters. The other coefficients can be interpreted similarly (Figure 1A). In population 2, CRP has a weak linear negative relationship with ‘Pred (L) FEV1’, ‘Pred (L) FVC’, ‘FEF 25–75 l/s (pred)’, and ‘Pred FEV1: FVC ratio’ (Figure 1B). Whereas CRP has a positive linear association with parameters like ‘Value (L) FEV1’, ‘Value (L) FVC, ‘FEF 25–75 l/s’, ‘% pred FEV1’, ‘% pred FVC’, and ‘% pred FEV1: FVC ratio’ (Figure 1B). TNF seems to have a linear positive association with variables like ‘Pred (L) FEV1’, ‘Pred (L) FVC’, ‘FEF 25–75 l/s (pred)’, ‘Value (L) FEV1’, and ‘Value (L) FVC’ (Figure 1C). The strength is medium for the first two variables, r = 0.50 and r = 0.53, respectively (Figure 1C). The figure below shows that TNF-alpha in population 2 has a weak negative linear relationship with almost all the parameters, except for ‘FEF 25–75 l/s’, where there is a very weak positive (r = 0.16) linear association (Figure 1D). In population 1, the variables like ‘Value (L) FEV1’, ‘Value (L) FVC’, ‘Value FEV1: FVC ratio’, ‘FEF 25–75 l/s’, ‘% pred FEV1’, ‘% pred FVC’, and ‘% pred FEV1: FVC ratio’ have a positive linear association with IL6, although the strength is weak (Figure 1E). In population 2, most parameters negatively associate with IL6 (Figure 1F). Only a few parameters like ‘Pred (L) FEV1’, ‘Pred (L) FVC’, and ‘FEF 25–75 l/s (pred)’ have a weak linear positive association with IL6 (Figure 1F).

## 4. Discussion

The main findings of this study were as follows: Serum levels of IL-6, IL-8, TNF-α, and CRP were similar between populations. Notably, we found that IL-8 and IL-1Ra were undetected in population 1, while IL-6 was seven times higher in both populations compared to the normal range. IL-8 was below the limit of detection (LOD) in population 1, while it was approximately 2.5 times higher in population 2 compared to the normal range. Meanwhile, TNF-α was 21 times higher in population 1 and 19 times higher in population 2 compared to the normal range. CRP was within the normal range and considering that most of the samples had an IL-1Ra level below the LOD, it is safe to assume that IL-1Ra was mostly undetected in population 1. In contrast, IL-1Ra was almost 1.5 times higher in population 2 compared to the normal range.

These findings are particularly noteworthy as the complex role of inflammation involving the release of pro-inflammatory cytokines, recruitment of immune cells to the site of infection [16], granuloma formation [17], and tissue necrosis [18] has been established. These processes are implicated in TB infection and the resulting tissue damage. During TB infection, sources of the pro-inflammatory cytokine IL-6 include macrophages and T cells [19]. IL-6 is important in initiating a T helper-1 response [19], which drives interferon-gamma production [20] to execute pro-inflammatory and antimicrobial effects. In instances of excessive IL-6 secretion, this leads to the increased collagen production by fibroblasts [21], contributing to lung damage. Findings by Fielding et al. (2014) propose an IL-6-mediated T helper-1 response that triggers the transition from acute to chronic inflammation, leading to tissue damage such as fibrosis [22]. The fact that IL-6 in both populations was higher than the normal range [20] was interesting because population 1 had completed their TB treatment more than a year ago, and population 2 was under TB treatment during this study. The high IL-6 in population 1 is concerning because it may suggest a persistent pro-inflammatory state, despite having completed their TB treatment such a long time ago. In turn, the high IL-6 in population 2 could be due to not having completed the entire treatment regimen yet. Taken together, the IL-6 data are suggestive of a persistent pro-inflammatory state post-TB (seeing that the TB has clinically resolved), at least in population 1, which could predispose these patients to several post-TB sequelae, like pulmonary hypertension. However, the latter is outside the scope of this current study.

As a defence against MTB infection, phagocytosis is mediated by macrophages and stimulates the secretion of IL-8 [23], which is a chemotactic factor [24] and contributes to granuloma formation [25]. The IL-8 actively recruits more neutrophils to the site of infection, which contributes to the excessive production of free radicals and the ultimate destruction of the lung parenchyma. The undetected IL-8 in population 1 could be because of successful TB treatment. At the same time, this was higher than normal [20] in population 2, possibly because they were ill with severe TB despite being on treatment. These data suggest that TB treatment effectively reduces IL-8 but not IL-6. This could speak to the extent to which different cytokines play a role in TB infection and may indicate that in follow-up studies, IL-6 is most likely the biomarker to use.

TNF-α was 21 times higher in population 1 and 19 times higher in population 2 compared to the normal range [20]. TNF-α is pivotal in eliciting an effective immune response against MTB infection [26] and is produced by activated macrophages [27]. This pro-inflammatory marker is considered pro-fibrotic as it induces the proliferation of fibroblasts [28]. TNF-α further stimulates the release of free radicals, namely reactive oxygen species and reactive nitrogen species, to cause parenchymal destruction and cavitation [29]. Moreover, the role of TNF-α extends to the preservation of the granuloma structure [30], a hallmark of TB [17], which restricts MTB dissemination [31]. Conversely, excessive TNF-α production is harmful as it facilitates necrosis [32] and cavitation, which impairs lung function [33]. This is evident in another study [34], which noted an increased TNF-α concentration following 7 days of TB treatment that correlated with clinical deterioration. Another study [35] established a correlation between increased TNF-α concentrations and enlargement in cavity size. Therefore, our study confirms the key role of TNF-α and highlights that this remains unchanged one year post-TB, despite treatment completion.

IL-1Ra was undetected in population 1. The reasons for this are unclear; however, it could be that the concentration of IL-1Ra in the samples of population 1 was below the LOD (10 ng/mL) of the kit or that TB treatment was effective in maintaining IL-1Ra at low levels [15]. IL-1Ra belongs to the IL-1 family of cytokines, which includes IL-1a and IL-1b [36]. IL-1a and IL-1b are agonists that function as pro-inflammatory cytokines and are involved in immune cell recruitment during TB infection [37]. In contrast, IL-1Ra assumes the role of an antagonist by competing for the same receptor [38]. However, IL-1Ra does not trigger an intracellular response and is an indigenous anti-inflammatory cytokine in various diseases, including granulomatous pulmonary diseases [39]. The low IL-1Ra in population 1, compared to the normal range of this marker [15], could also suggest that their IL-1 was lower too; however, this is speculation, as we did not measure IL-1 directly. The elevated IL-1Ra concentrations observed in population 2 suggest a potential elevation of IL-1 in TB patients, possibly due to active TB infection. Conversely, the absence of IL-1Ra in population 1 indicates a potential dampening of the immune response, likely contributing to the normalization of IL-1 concentrations post-TB treatment. Our findings agree with observations made by Su et al. (2010) of a significant reduction in plasma IL-1b in patients with pulmonary TB after 2 months of TB treatment, further validating the absent IL-1Ra concentration in population 1 [40]. It is important to note that we did not measure IL-1, making this speculation at this point. However, previous research [37] has shown a key role of IL-1 in TB. Regardless, our finding suggests that IL-Ra is high in active drug-sensitive TB because the bacterium is not yet under check, and the treatment regimen is not completed.

CRP is an acute-phase protein that is increased in adults with TB, although whether it directly contributes to lung damage in TB remains elusive. CRP was approximately 49 times higher in both populations, compared with the normal range [14]. This makes sense for population 2, as they had severe TB despite receiving in-hospital treatment, and they generally had poorer lung function and had more than one previous TB episode. Thus, the active infection is likely responsible for the high CRP. For population 1, the excessively high CPR is confusing, as these patients have resolved TB symptoms, have good lung function, and mostly had only one or a maximum of two previous TB episodes. This suggests that despite successful TB treatment, they have a pro-inflammatory profile that may indicate other possible underlying conditions unrelated to TB, or it could mean that the TB-induced CRP peak does not clear post-TB. If the latter is true, it is concerning, as perpetually high CPR may contribute to other complications in the pulmonary circulation and other organs.

The patients in population 1 completed TB treatment more than one year before enrollment. Regarding their TB stage and lung function, they had 1–2 previous TB episodes and relatively good lung function (considering the FVC % predicted and FEV 1% predicted data). Yet, regarding their inflammatory biomarkers, IL-6, TNF-α, and CRP were higher than the normal ranges. This suggests that despite the relatively good lung function post-treatment, their pro-inflammatory state remains high more than one year after treatment completion. One could hypothesize that smoking could be responsible for this pro-inflammatory state. Still, it may also be the result of a previous TB infection or other confounding factors not measured in this cohort, like air pollution. Population 2 has active TB and received in-hospital treatment for severe TB. Regarding their TB stage and lung function, they had 2–8 previous TB episodes and relatively poorer lung function (considering the FVC % predicted and FEV 1% predicted data). In terms of their inflammatory biomarkers, IL-6, TNF-α, CRP, and IL-1Ra were higher than the normal ranges. Their lung function is most likely poor due to the severe TB, but it could also be because they have had 2–8 previous TB episodes.

We tested for normality before performing the Pearson correlation. We know that the Pearson correlation coefficient measures the degree of linear relationship. So, whenever we receive large values closer to +1 or −1, there is no question at ALL that the association is linear. The fact that the Pearson correlation is close to zero may not necessarily indicate that the association is absent. The fact that the Pearson correlation is close to zero may indicate two things: either the association between the two variables is lacking, or there might be a nonlinear relationship between the two variables. The correlation coefficient would not be as significant if a linear association were absent.

In population 1, variables like FEV1, FVC, FEV1: FVC ratio, FEF 25–75 l/s, % pred FEV1, % pred FVC, and % pred FEV1: FVC ratio display a positive linear association with IL6, although the strength is weak. This may suggest that as the lung function in population 1 increases, so does IL-6. This highlights the key role of IL-6 in TB, but also that despite completion of TB treatment and better lung function, IL-6 remains high, which speaks to the possible need for continued anti-inflammatory therapy post-TB. In population 2, most parameters have a negative linear association with IL-6. Only a few parameters like Pred (L) FEV1, Pred (L) FVC, and FEF 25–75 l/s (pred) have a weak linear positive association with IL6. Regardless, this suggests that IL6 plays a vital role as a biomarker in the context of the disease. Specifically for population 2, it means that severe TB and poor lung function, together with 2–8 previous TB episodes, are associated with high IL-6.

The level of TNF-α was higher in population 1, and in addition, TNF-α has a linear positive association with Pred (L) FEV1, Pred (L) FVC, FEF 25–75 l/s (pred), FEV1, and FVC in population 1. The correlation data suggests that as lung function increases, the TNF-α levels rise accordingly. Therefore, despite the relatively better lung function one year after the completion of TB treatment, if anti-inflammatory treatment regimen is not started, the TNF-α would most likely continue to increase to a maximum, which could contribute to severe long-term lung damage. In population 2, TNF-α was also higher than the normal range and displayed a weak negative linear relationship with almost all the parameters, except for FEF 25–75 l/s, where there is a very weak positive linear association, suggesting that TNF-α is linked with lung function in population 2. Furthermore, it indicates that as the lung function reduces in this population, TNF-alpha increases accordingly. Again, this confirms that TNF-α is a promising biomarker to study lung function in the post-TB stages (i.e., more than one year after the completion of TB treatment and/or 2–8 previous TB episodes). TNF-α is likely a key player in patients with active and treated, severe TB. This could be due to the active TB infection, but may also highlight the need to manage the pro-inflammatory state in these patients despite being on TB drugs.

In population 1, CRP has a weak linear negative relationship with all the lung function parameters; whereas, in population 2, CRP only displays a weak linear negative relationship with Pred (L) FEV1, Pred (L) FVC, FEF 25–75 l/s (pred), and Pred FEV1: FVC ratio. In terms of population 1, this suggests that although CRP was higher than the normal range, it was not a promising biomarker for Pred (L) FEV1, Pred (L) FVC, FEF 25–75 l/s (pred), and Pred FEV1: FVC ratio. In addition, in population 2, CRP also displayed a positive linear association with parameters like FEV1, FVC, FEF 25–75 l/s, % pred FEV1, % pred FVC, and % pred FEV1: FVC ratio, making its usefulness questionable in this context. Taken together, it is concerning that CRP remains high post-TB (despite treatment); however, the correlation data suggest that it better reflects changes in FEV1, FVC, FEF 25–75 l/s, % pred FEV1, % pred FVC, and % pred FEV1: FVC ratio, as opposed to the other parameters in spirometry.

The study remains purely observational, and the influence of potential confounding factors like obesity, autoimmune diseases, or other infections/inflammations present at the time of evaluation, as well as HIV status, should be considered when drawing inferences from inflammatory data for population 2, where HIV was relatively more common. One could argue that HIV was relatively high in population 2, which could contribute substantially to the higher levels of inflammatory markers. However, these markers were also elevated in population 1, where HIV was extremely low in prevalence. In addition, all HIV patients were on therapy. Therefore, the elevated pro-inflammatory state in the post-TB context is most likely due to TB itself. There may have been other confounding factors that were not studied due to the intrinsic design of the parent study, and these include, but are not limited to, auto-immune diseases, other infections, and vaccinations.

Remember that the goal is not to statistically compare the two populations but to observe their inflammatory profiles in different stages of the disease continuum. For example, in population 2, IL-6, IL-8, and TNF-α were higher than the normal range for these markers. Considering that they had active and treated TB at the time of blood collection, the high inflammation could be due to active TB infection or other factors like HIV or possible co-morbidities. This is as far as the interpretation of these findings can go, given the limitations of the sub-study. For population 1, on the other hand, TB was resolved, treatment was successful, and only one person had HIV, while no one had heart disease, although 15 were current smokers. In this case, smoking might also be a contributor to the fact that IL-6 and TNF-α were higher than the normal range. Although the correlation data supports the notion that there is a relationship between pro-inflammatory markers and impaired lung function, the data might not be perfect (no data are). Still, it does imply the essential roles of inflammation in these two stages of TB (one-year post-TB and current TB). This points out the need for better and larger studies that can delineate the involvement and possible targeting of inflammation during and after TB resolution.

## 5. Conclusions

We demonstrate a potentially unique role for IL-8 and IL-1Ra in patients one year post-TB, whereas this effect is not observed in patients with active TB. We also show that IL-6, IL-8, TNF-α, and CRP are key cytokines to consider in combating persistent inflammation post-TB. These may be attributed to multiple factors, including persistent infection, abnormal tissue repair, and variations in the host immune system that trigger an immune response despite the completion of TB treatment. This calls for clinicians to consider anti-inflammatory therapies in reducing post-TB inflammation. Our findings highlight the importance of targeted therapeutic approaches for IL-1Ra, with potential relevance to IL-1 in effectively addressing TB. We, therefore, propose that future studies must investigate the true efficacy of TB drug regimens, and our findings might highlight a need for adjunct therapies with anti-inflammatory actions.

## Figures and Tables

**Table 1:** Descriptive patient data of the population characteristics.

	Population 1 (n = 22)	Population 2 (n = 21)
Age (years) n (%)	41.09 ± 14.6	35.33 ± 9.9
18–29	6 (27.3)	7 (33.3)
30–39	5 (22.7)	7 (33.3)
40–49	5 (22.7)	6 (28.6)
50–59	4 (18.2)	1 (4.8)
≥60	2 (9.1)	0 (0.0)
Sex, n (%)		
Female	9 (40.9)	9 (42.9)
Male	13 (59.1)	12 (57.1)
Smoking status, n (%)		
Current	15 (68.2)	9 (43.0)
Ex-smoker	1 (4.5)	4 (19.0)
Never	6 (27.3)	4 (19.0)
Undisclosed	0 (0.0)	4 (19.0)
HIV status, n (%)		
Positive	1 (4.5)	9 (42.9)
Negative	19 (86.4)	12 (57.1)
Unknown	2 (9.1)	0 (0.0)
Known Heart Disease, n (%)		
Yes	0 (0.0)	3 (14.3)
No	22 (100.0)	18 (85.7)

**Table 2:** The two populations’ clinical data (TB episodes recorded for each patient and spirometry parameters).

TB Episodes	Population 1 (n = 22) n (%)	Population 2 (n = 21) n (%)
0	0 (0.0)	8 (38.1)
1	11 (50.0)	3 (14.3)
2	11 (50.0)	3 (14.3)
3	0 (0.0)	5 (23.8)
>3	0 (0.0)	2 (9.5)
Spirometry		
FVC (L)	3.7 (0.95)	3.7 (0.54)
FVC % (predicted)	76.4 (14.69)	59.4 (21.80)
FEV1 (L)	2.1 (0.78)	1.5 (0.67)
FEV 1% (predicted)	67.8 (18.64)	48.6 (23.05)
FEV1/FVC	60% (0.82)	41% (1.24)
FEF 25–75	3.1 (0.78)	3.3 (0.53)

Abbreviations: FVC, forced vital capacity; FEV1, forced expiratory volume in 1 s; FEF, forced expiratory volume. The spirometry parameters are presented as mean and SD values.

**Table 3:** Inflammatory markers of populations 1 and 2.

Inflammatory Biomarker	Population 1	Population 2	Normal Range
IL-6 (ng/mL)			
Mean	9.518	13.98	5.36 [13]
SD	6.206	22.42	
SEM	1.388	5.013	
Samples > LOD	20	20	
IL-8 (ng/mL)			
Mean	9.959	31.75	12.86 [13]
SD	44.54	63.35	
SEM	9.959	14.17	
Samples > LOD	1	8	
TNF-alpha (ng/mL)			
Mean	92.68	86.93	4.36 [13]
SD	46.43	50.81	
SEM	10.38	11.36	
Samples > LOD	20	20	
CRP (mg/dL)			
Mean	14.98	13.73	<0.3 [14]
SD	2.126	2.808	
SEM	0.4753	0.6279	
Samples > LOD	20	20	
IL-1Ra (ng/mL)			
Mean	<LOD	247.5	190 [15]
SD	<LOD	327.1	
SEM	<LOD	73.14	
Samples > LOD	0	11	

## Data Availability

The availability of patient-related data is limited due to privacy and ethical restrictions.
